# The Shift of Thermoneutral Zone in Striped Hamster Acclimated to Different Temperatures

**DOI:** 10.1371/journal.pone.0084396

**Published:** 2014-01-06

**Authors:** Zhi-Jun Zhao, Qing-Sheng Chi, Quan-Sheng Liu, Wei-Hong Zheng, Jin-Song Liu, De-Hua Wang

**Affiliations:** 1 College of Life and Environmental Science, Wenzhou University, Wenzhou, Zhejiang, China; 2 State Key Laboratory of Integrated Management for Pest Insects and Rodents, Institute of Zoology, Chinese Academy of Sciences, Beijing, China; 3 Guangdong Key Laboratory of Integrated Pest Management in Agriculture, Guangdong Entomological Institute, 105 Xin’gang Xilu, Haizhu, Guangzhou, China; St. Joseph's Hospital and Medical Center, United States of America

## Abstract

Temperature affects all biological functions and will therefore modulate ecologically significant interactions between animals and their environment. Here, we examined the effect of ambient temperature (T_a_) on the thermal biology and energy budget in striped hamsters acclimated to cold (5°C), warm (21°C) and hot temperatures (31°C). Thermoneutral zone (TNZ) was 22.5–32.5°C, 25–32.5°C and 30–32.5°C in the cold-, warm- and hot-acclimated hamsters, respectively. The cold acclimation decreased the lower critical temperature and made the TNZ wider, and hot exposure elevated the lower critical temperature, resulting in a narrow TNZ. Within the TNZ, cold-acclimated hamsters showed a significantly higher rate of metabolism and thermogenesis than those acclimated to hot temperature. Digestive enzymes activities, including intestinal sucrase, maltase, L-alanine aminopeptidase-N and leucine aminopeptidase were higher in the cold than in the hot. The changes in metabolic rate and thermogenesis at different temperatures were in parallel with cytochrome c oxidase activity and uncoupling protein 1 gene expression of brown adipose tissue. This suggests that the shift of the lower critical temperature of TNZ is possibly associated with the rate of metabolism and thermogenesis, as well as with the digestive capacity of the gastrointestinal tract at different T_a_. The upper critical temperature of TNZ may be independent of the changes in T_a_. The changes of lower critical temperature of TNZ are an important strategy in adaption to variations of T_a_.

## Introduction

Temperature affects all biological functions and will therefore modulate ecologically significant interactions between animals and their environment [1]–[3]. Proper adjustments of the thermoregulatory mechanisms of animals definitely increase the capacity to survive the climate changes in their natural environment [4]–[9]. The rates of metabolism and thermogenesis are important physiological adjustments of animals in response to the ambient temperature (T_a_) changes. Animals that are acclimated to winter or winter-like conditions usually show significant increases in basal or resting metabolic rate (BMR or RMR), which are paralleled with the increased energy intake, and are also associated with the morphological and functional changes in visceral organs and digestive system [5]. The increased energy spent for thermoregulatory thermogenesis in cold-acclimated animals are mainly employed to maintain stable body temperature (T_b_) when the difference between T_a_ and T_b_ becomes large [3], [10], [11]. Actually, it has been observed that T_b_ of small mammals is surprisingly variable on daily or seasonal patterns, which is partly due to the daily and seasonal changes in T_a_ [3], [9], [12]–[14]. Thus an animal could gain an advantage by regulating T_b_ to a lower level at cold temperatures because heat loss would be decreased due to reduced difference between T_b_ and T_a_ [3], [14].

We have previously found a disagreement in the literature on thermoregulatory characteristics of striped hamsters (*Cricetulus barabensis*). Song and Wang (2003) reported that they had relatively high T_b_, narrow thermal neutral zone (TNZ) and high metabolic rate [15]. Inconsistently, Liu et al. (2003) found a relatively low T_b_, wider TNZ and high metabolic rate [16]. Zhao and his colleague (2010) argued that this species had seasonal fluctuations in metabolic rate and thermal conductance, by which narrower TNZ with higher upper critical temperature in summer enabled hamsters to survive the hot weather, and wider TNZ with higher metabolic thermogenesis likely increased the capacity to cope with cold winter [14], [17]. It has been also reported that other animals living cold conditions have relatively low lower critical temperatures and show extremely wide TNZ than those from warm conditions [18]–[20]. This may indicate that a shift of TNZ occurs due to the changes in T_a_, suggesting a potential role of T_a_.

Adaptive nonshivering thermogenesis (NST) is known to be mediated by brown fat-specific uncoupling protein 1 (UCP1), which uncouples oxidative metabolism from mitochondrial ATP synthesis [21]. Cytochrome c oxidase (complex IV) in mitochondria of brown adipose tissue is involved in mitochondrial energy metabolism [22]. Animals that are exposed to the temperatures below their TNZ increase metabolic thermogenesis, which is in parallel with upregulation of ucp1 gene expression and also cytochrome c oxidase activity of brown adipose tissue [12], [21], [23]–[28]. Thus, adaptive adjustments of brown adipose tissue mitochondrial ucp1 gene expression and cytochrome c oxidase activity may be one of the possible mechanisms mediating the shift of TNZ at different T_a_.

The striped hamster, a nonhibernating animal, is a principal rodent in northern China and is also distributed in Russia, Mongolia, and Korea [29]. The characteristics of ecological physiology and the habitat are well described previously [14], [15], [17], [29], [30]. Briefly, this species is subjected to marked seasonal fluctuations in T_a_ since the climate is arid and characterized by warm and dry summer with maximum temperature of 42.6 °C and cold winter with minimum temperatures below −20 °C [14], [17], [29]. Significant differences in metabolic thermogenesis and TNZ were observed in seasonal acclimated hamsters [14], [15], [17], [30]. In the present study, we aimed to examine the changes in metabolic thermogenesis, digestive enzymes and cytochrome c oxidase activity and ucp1 gene expression of brown adipose tissue over a range of ambient temperatures in striped hamster acclimated to warm, cold or hot conditions. We tested the hypotheses that shift of TNZ, along with adjustments of metabolic thermogenesis, was an important strategy in adaption to climate change. Further, adaptive regulations of digestive enzymes, cytochrome c oxidase activity and UCP1 gene expression of brown adipose tissue would be involved in shift of TNZ.

## Methods and Materials

### Ethics statement

All experimental procedures were in compliance with the Animal Care and Use Committee of Wenzhou University. This study was approved by the Committee (11-1029-052).

Striped hamsters were obtained from our laboratory-breeding colony, which started with animals that were initially trapped from farmland at the center of the Hebei province (115°13′E, 38°12′S), North China Plain. This breeding colony was maintained at 23±1°C under a 12L:12D (light: dark, lights on at 0800h) photoperiod. Food (standard rodent chow; produced by Beijing KeAo Feed Co., Beijing, China) and water were provided *ad libitum*. Adult female hamsters (3.5 – 4 months old in the breeding colony) were kept singly and used in this study.

**Experiment 1** was designed to examine the effects of cold and hot acclimation on thermal biology and digestive enzymes of small intestine. Seventy two hamsters were randomly assigned to the warm, cold and hot group (three groups), during which hamsters were maintained at 23°C (Warm, *n* = 24), acclimated to 5°C (Cold, *n* = 24) and 31°C (Hot, *n* = 24) for six weeks, respectively.

### Metabolic rate

Metabolic rate was quantified as the rate of oxygen consumptions using an open-flow respirometry system (Sable systems, USA) at the end of the acclimation. In detail, air was pumped at a rate of 600–850 ml/min through a cylindrical sealed Perspex chamber, which was put in an incubator to control the chamber temperature within ±0.5 °C. Gases leaving the chamber were dried using anhydrous silica gel and directed through the oxygen analyzer (FC-10, Sable systems, USA) at a flow rate of 150–175 ml/min. The data were collected every 10 s by an analogue-to-digital converter (STD-UI2, Sable system). Animals were fasted for 4 hours and transferred into chambers for 1 hour for adaptation to the chamber. metabolic rate measurements were done separately for 2 hrs for each temperature, which started with 37.5°C, and then 35, 32.5, 30, 27.5, 25, 22.5, 20, 15, 10 and 5°C on a two-day interval. All measurements were performed between 10:00-17:00 to correct for a possible effect of the circadian rhythm. Metabolic rate was calculated from the consecutively lowest readings over 5 min at each temperature, using the following equation: VO_2_ = FR (FiO_2_-FeO_2_)/(1-FiO_2_×(1-RQ)), where FR is the flow rate, FiO_2_ is input fractional concentration of O_2_ to the chamber, FeO_2_ is excurrent fractional concentration of O_2_ from the chamber, and RQ is respiratory quotient [31]–[33]. Here, RQ was assumed to be 0.85 [32], [34].

### Body temperature (T_b_) and thermal conductance

T_b_ was recorded after each metabolic rate test at each temperature. Rectal temperature (hereafter referred to as T_b_) of animals was measured, using a digital mouse thermometer (produced by the Beijing Normal University). The probe of the thermometer was inserted 3 cm into the rectum and a reading was taken after 30 s. Thermal conductance is expressed as wet thermal conductance (*C*_wet_), which is the rate of heat loss relative to the thermal gradient, uncorrected for evaporative heat loss. *C*_wet_ was calculated using the following equation: *C*_wet_ (mlO_2_/g·h·°C)  =  metabolic rate (mlO_2_/g·h) / (*T*_b_-*T*_a_) (°C). Where, *C*_wet_ is the “wet” thermal conductance; metabolic rate is the rate of metabolism at each temperature; T_b_ is the body temperature of animals and T_a_ is the ambient temperature. *C*_wet_ is calculated over a range from 5–30°C, but excludes 32.5 and 35°C because this formula is acceptable only at moderate to low temperatures, where evaporative heat loss is a small fraction of total heat loss [17], [20], [35]–[40].

### Cytochrome c oxidase activity of brown adipose tissue and visceral organs

Animals were sacrificed by decapitation. Scapular brown adipose tissue and visceral organs (the liver, heart, lung, spleen and kidneys) were quickly removed, weighed (to 1 mg), and stored in liquid nitrogen for the later cytochrome c oxidase measurements. The tissues were homogenized and mitochondria protein was prepared as described previously [41], [42]. Mitochondrial protein concentration was determined by the Folin phenol method with bovine serum albumin as standard [43]. Cytochrome c oxidase activity of the whole tissue was measured polarographically with oxygen electrode units (Hansatech Instruments LTD., England) [14], [42], [44].

### Digestive enzyme activity

Small intestine was separated and removed shortly after brown adipose tissue removal. As described previously [45], duodenum, jejunum and ileum were removed and weighed separately without the contents and then immediately preserved in liquid nitrogen for the enzyme assays. The digestive tracts were homogenized in 0.9% NaCl solution (1∶10, w/v) using an electric glass homogenizer, during which temperature was controlled in an ice-water bath. We measured the activity of the enzymes in whole-tissue homogenates rather than in mucosal samples to avoid underestimation of activity as previously reported [46], [47].

Sucrase (E.C. 3.2.1.48) and maltase (E.C. 3.2.1.20) activity were measured using a commercial kit (produced by Jiancheng Biotech Co. Ltd., Nanjing, China) according to the instruction. Sugar and maltose were used as the substrate for sucrase and maltase activities analysis, respectively. Briefly, 10 µl of the homogenate was added to 20 µl of assay mix (reagent buffer no. 1) in each tube, vortexed, and incubated in a water bath at 37°C for 20 min. We terminated the reaction by adding 10 µl of an ending solution (reagent no. 2) to each tube followed by 1 ml detective reagents solutions (reagent no. 3). The absorbance was read at 505 nm. Activity was expressed in U per min (1 U was defined as 1 nmol sucrase or maltase hydrolyzed at 37°C by 1 mg tissue protein per minute). L-alanine aminopeptidase-N (AAP, E.C. 3.4.11.2) and leucine aminopeptidase (LAP, EC 3.4.11.1) activity were measured using AAP and LAP assay kit, respectively (Huili Biotech Co. Ltd., Changchun, China) according to the instruction. Briefly, L-alanine-p-nitroanilide and L-leucine-p-nitroanilide were used as the substrates, respectively. 10µl of the homogenate was added to 0.5 ml of assay mix. The absorbance was measured at 405 nm, and activity was also presented in U/min.

**Experiment 2** was performed to examine the changes in thermogenesis, energy intake and digestive enzymes, as well as UCP1 gene expression of brown adipose tissue in response to hot-to-cold or cold-to-hot transitions. One hundred and twenty eight hamsters were randomly acclimated to either cold (5°C, *n* = 64) or hot condition (31°C, *n* = 64) for ten weeks. Then, animals in the cold regiment were randomly assigned into four groups (*n* = 16 of each group): cold group, animals were continued to be kept at 5°C, and to-hot-2d, 14d and 40d groups, within which animals were transferred into the hot condition (31°C) for 2, 14 and 40 days, respectively. Similarly, animals at the hot were also assigned into four groups (*n* = 16 of each): hot group, animals were continued to be kept at 31°C, and to-cold-2d, 14d and 40d groups, animals were transferred into the cold condition (5°C) for 2, 14 and 40 days, respectively. Here were total eight groups arranged.

### Metabolic rate and maximal nonshivering thermogenesis (NST_max_)

Metabolic rate was measured in hamsters after the end of each temperature transition. The measurements were performed using the methods mentioned above, but instead the chamber temperature was controlled at 25, 27.5, 30 and 32.5°C, respectively. On the day next to metabolic rate measurements, NST_max_ was quantified as the maximum rate of oxygen consumptions in response to norepinephrine (NE) [23]. NST_max_ was induced by a subcutaneous injection of NE at 25±0.5°C. The mass-dependent dosage of NE (Shanghai Harvest Pharmaceutical Co. LTD) was calculated according to the equation: NE (mg·kg^−1^)  = 6.6 Mb^−0.458^(g) (Mb, body mass) [48]. NST_max_ was calculated according the same methods with metabolic rate, but instead used two continuous stable maximal recordings [33], [42].

### Energy intake and digestibility

During the last two days of each experimental regiment, energy intake and digestibility were measured as described previously [10]. Briefly, food was provided quantitatively, and food residues left on the hopper and mixed with bedding, as well as feces were collected from each animal over 2 days. Both food residues and feces were separated manually after they were dried at 60°C to constant mass. The gross energy contents of the food and feces were determined using a Parr 1281 oxygen bomb calorimeter (Parr Instrument, Moline, IL, USA). Dry matter intake, gross energy intake, digestible energy intake, and apparent energy assimilation efficiency (digestibility) were calculated as follows [4], [10]: Dry matter intake (g/d) = food intake (g/d) × dry matter content of food (%) - dry spillage of food (g/d); Gross energy intake (kJ/d) =  Dry matter intake (g/d) × gross energy content of food (kJ/g); Digestible energy intake (kJ/d) =  gross energy intake - [dry feces mass (g/d) × gross energy content of feces (kJ/g)]; Digestibility (%) = digestible energy intake / gross energy intake ×100%.

### Real-time RT-QPCR analysis

Animals were sacrificed by decapitation. Scapular brown adipose tissue was quickly removed and stored in liquid nitrogen for RT-QPCR analysis. Total RNA was isolated from brown adipose tissue using the Trizol method (TAKARA, China) according to the manufacturer's protocol. First-strand cDNA synthesis was performed on 2 µg of total RNA with AMV Reverse Transcriptase (TAKARA, China) using random primer Oligo(dT)_18_. 2µl of the reverse transcription reaction was used as a template for the subsequent PCR reaction using gene-specific primers (UCP1, sense: 5′-GGGACCATCACCACCCTGGCAAAAA-3′ and antisense, 5′-GGCTTTCTGTTGTGGCTAT-3′, predicted production was 330 bp). The quantitative analysis was performed using the Mx3000P Real-Time QPCR system (Stratagene, La Jolla, CA, USA), and the reactions were done using the SYBR Green PCR master mix kit following manufacturer's instructions. Reactions for UCP1 gene expression were carried out for 95°C, 30 s and 40 cycles at 95°C for 5 s, 55°C for 30 s and 72°C for 30 s, and then followed by a dissociation stage analysis. The reaction consisted of SYBR Premix EX Tag TM (2 ×), 12.5µl; 3′ primers (10µM), 0.5µl; 5′ primers (10µM), 0.5µl; ROX Reference Dye II (50 ×), 5µl and template cDNA, 2µl β-actin (sense: 5′- CGGGACCTGACAGACTAC-3′ and antisense: 5′- CTCGTTGCCAATGGTGAT -3′, 213 bp) was used as a reference gene.

### AAP and LAP activity

After brown adipose tissue removed, small intestine was separated and homogenized as described above. Since the effect of temperature on the digestive enzyme activity was similar between duodenum, Jejunum and Ileum, AAP and LAP activity were totally measured in whole small intestine instead of separately in duodenum, Jejunum and Ileum. Measurements of AAP and LAP activity were performed according to the protocol described in the “Digestive enzyme activity” section.

### Statistics

Data were analyzed using SPSS 13.0 statistic software. Experiment 1, repeated-measures analysis of variance (RM-ANOVA) was used to determine the changes in metabolic rate, T_b_ and thermal conductance between the temperatures ranging from 5 to 37.5 °C. Differences in metabolic rate, T_b_ and thermal conductance between hot, warm and cold groups were examined using one-way ANOVA. Cytochrome c oxidase activity of brown adipose tissue, masses of visceral organs, mitochondria protein (MP) content and digestive enzymes were examined using one-way ANCOVA, with carcass mass as a covariate where required. In experiment 2, changes in metabolic rate from 25 to 32.5 °C were analyzed using RM-ANOVA. Differences in metabolic rate, NST_max_, energy budget, AAP and LAP activities and UCP1 gene expression between the cold, to-hot-2d, -14d and -40d groups or between the hot, to-cold-2d, -14d and -40d groups were examined using one-way ANOVA, followed by Tukey post-hoc tests. Data were presented as means ± s.e.m. The statistic significance was determined at *P*<0.05.

## Results

Effects of cold and hot acclimation on thermal biology and digestive enzymes of small intestine

### Metabolic rate

**Figure 1:**
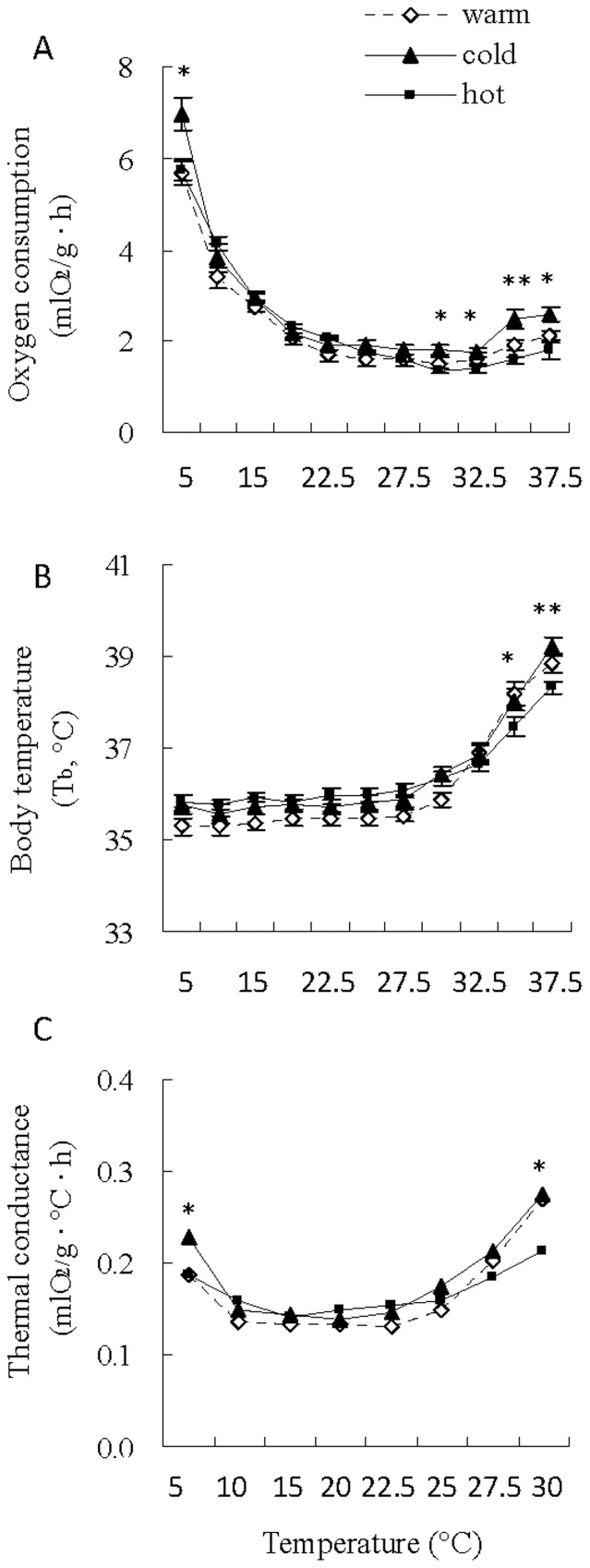
Oxygen consumption (A), body temperature (B) and thermal conductance (C) over a range of temperature between 5 and 37.5°C in striped hamsters acclimated to warm (23°C), cold (5°C) and hot (31°C) conditions. *, significant difference between the three groups (*P*<0.05), **, *P*<0.01. Data are means ± s.e.m.

Hamsters that were kept at the warm condition (23°C) showed significant decreases in metabolic rate from 5 to 20°C (*F*_3,33_ = 108.03, *P*<0.001), and then maintained stable metabolic rate from 22.5 to 32.5°C (*F*_4,44_ = 0.59, *P*>0.05), during which metabolic rate averaged 1.60±0.13 mlO_2_/g · h (Fig. 1A). This indicated that TNZ was 22.5 – 32.5°C in hamsters that were acclimated to the warm condition. For hamsters acclimated to the cold condition (5°C), metabolic rate decreased significantly from 5 and 22.5°C (*F*_4,44_ = 137.25, *P*<0.05) and reached a lower and stable level between 25 and 32.5°C (*F*_3,27_ = 2.25, *P*>0.05), during which the mean metabolic rate was 1.82±0.10 mlO_2_/g/h, suggesting a TNZ of 25– 32.5°C. Significant reductions in metabolic rate were observed from 5 to 27.5°C in hamsters acclimated to the hot condition (*F*_6,66_ = 216.87, *P*<0.001), and metabolic rate between 30 and 32.5°C was not statistically different, indicating a narrow TNZ of 30 – 32.5°C (*F*_1,10_ = 3.10, *P*>0.05). Within the TNZ hot hamsters showed minimum metabolic rate (1.36±0.06 mlO_2_/g · h). When chamber temperature increased above 32.5°C, metabolic rate increased significantly in the three groups. At 5°C and 30, 32.5, 35 and 37.5°C, cold hamsters showed significantly higher metabolic rate than warm and hot hamsters (Fig. 1A).

### Body temperature (T_b_)

Hamsters showed significant increases in T_b_ from 5 to 37.5°C (warm, 35.3±0.2°C to 38.9±0.1°C, *F*_10,100_ = 57.47, *P*<0.001; cold, 35.8±0.2°C to 39.2±0.2°C, *F*_10,90_ = 66.38, *P*<0.001; hot, 35.8±0.1°C to 38.3±0.1°C, *F*_10,100_ = 57.67, *P*<0.001, Fig. 1B). T_b_ differed significantly between warm, cold and hot groups at 35°C (*F*_2,33_ = 3.96, *P*<0.05) and 37.5°C (*F*_2,33_ = 6.57, *P*<0.01, Fig. 1B), but the group differences were not significant at 5, 10, 15, 20, 22.5, 25, 27.5, 30 and 32.5°C, respectively.

### Thermal conductance

Significant changes in thermal conductance were observed over the range of 5 to 30°C, during which thermal conductance decreased significantly from 5 to 15°C, and then increased after temperature elevated above 20°C and reached a maximum at 30°C (warm, *F*_7,70_ = 25.93, *P*<0.001; cold, *F*_7,63_ = 49.27, *P*<0.001; hot, *F*_7,70_ = 20.03, *P*<0.001, Fig. 1C). Cold hamsters showed higher thermal conductance than warm and hot hamsters at 5°C (*F*_2,33_ = 4.11, *P*<0.05). Difference between the three groups was also observed at 30°C, where thermal conductance in hot group was significantly lower than warm and cold groups (*F*_2,29_ = 4.24, *P*<0.05, Fig. 1C).

### Masses and cytochrome c oxidase activity of brown adipose tissue and visceral organs

There were no differences in body mass and carcass mass between the three groups (Table 1). Cold hamsters showed significantly higher cytochrome c oxidase activity of brown adipose tissue, liver, spleen and kidneys than warm and hot hamsters (Table 1). Liver, heart and kidneys were significantly heavier in cold hamsters than that in warm and hot hamsters (Table 1).

**Table 1:** Masses and cytochrome c oxidase (COX) activity of brown adipose tissue and visceral organs in striped hamsters acclimated to warm, cold and hot conditions.

	warm (23°C)	cold (5°C)	hot (31°C)		
	*n* = 11	*n* = 11	*n* = 10	*F*	*P*
Body mass (g)	25.02±1.02	26.20±0.64	26.50±1.05	0.74	ns
Carcass mass (g)	17.03±0.82	16.62±0.43	18.33±0.75	1.76	ns
Brown adipose tissue					
Mass (g)	0.098±0.019	0.133±0.012	0.120±0.011	3.53	0.05
COX (nmol O_2_/min)	41.1±9.8^b^	63.1±7.6^a^	31.0±2.6^c^	6.25	**
Liver					
Mass (g)	1.015±0.118^b^	1.311±0.106^a^	0.977±0.114^b^	8.41	**
COX (nmol O_2_/min)	406.9±36.5^b^	523.9±68.6^a^	283.1±46.8^c^	5.24	*
Heart					
Mass (g)	0.127±0.004^b^	0.159±0.004^a^	0.115±0.007^b^	49.78	**
COX (nmol O_2_/min)	32.2±3.7	30.0±4.7	30.1±4.0	0.09	ns
Lung					
Mass (g)	0.219±0.012	0.222±0.007	0.207±0.017	0.72	ns
COX (nmol O_2_/min)	28.9±5.3	32.5±4.9	24.3±4.4	0.75	ns
Spleen					
Mass (g)	0.029±0.003	0.031±0.004	0.033±0.003	0.46	ns
COX (nmol O_2_/min)	4.1±0.9^b^	5.5±0.9^a^	2.5±0.5^c^	3.74	*
Kidneys					
Mass (g)	0.266±0.010^b^	0.325±0.016^a^	0.226±0.011^c^	31.35	**
COX (nmol O_2_/min)	178.1±18.3^b^	336.4±22.8^a^	144.1±15.0^c^	28.35	**

Data are means ± s.e.m. ns, non-significant difference; *, significant difference between the three groups (*P*<0.05), **, *P*<0.01. Different letters on the same row indicate significant difference (*P*<0.05).

### Masses and mitochondria protein content of small intestine

Cold hamster had heavier duodenum, jejunum and ileum than warm and hot hamsters (Table 2). Mitochondria protein contents of whole tissue of jejunum and ileum were higher in cold hamsters than that in warm and hot hamsters, whereas no differences were observed when they were corrected for tissue masses. Neither masses nor mitochondria protein contents differ between warm and hot hamsters (Table 2).

**Table 2:** Masses and mitochondria protein (MP) content of small intestine in striped hamsters exposed to warm, cold and hot conditions.

	warm (23°C)	cold (5°C)	hot (31°C)	*F*	*P*
Duodenum					
Mass (g)	0.167±0.009^b^	0.209±0.013^a^	0.145±0.014^b^	7.16	**
MP (mg/g)	93.89±4.89	96.89±4.68	88.83±5.55	0.65	ns
MP (mg in whole tissue)	15.99±1.78	20.43±2.34	13.35±2.24	2.81	0.08
Jejunum					
Mass (g)	0.180±0.011^b^	0.271±0.021^a^	0.172±0.010^b^	13.60	**
MP (mg/g)	113.10±4.76	117.64±4.77	115.20±3.18	0.28	ns
MP (mg in whole tissue)	19.91±1.67^b^	29.08±2.27^a^	19.54±1.24^b^	9.22	**
Ileum					
Mass (g)	0.146±0.014^b^	0.254±0.021^a^	0.135±0.009^b^	17.17	**
MP (mg/g)	109.26±5.22	122.40±5.27	112.20±3.55	2.11	ns
MP (mg in whole tissue)	17.23±1.70^b^	29.53±3.79^a^	15.20±1.12^b^	10.37	**

Data are means ± s.e.m. ns, non-significant difference; **, significant difference between the three groups (*P*<0.01). Different letters on the same row indicate significant difference (*P*<0.05).

### Activities of sucrase, maltase, AAP and LAP

**Figure 2:**
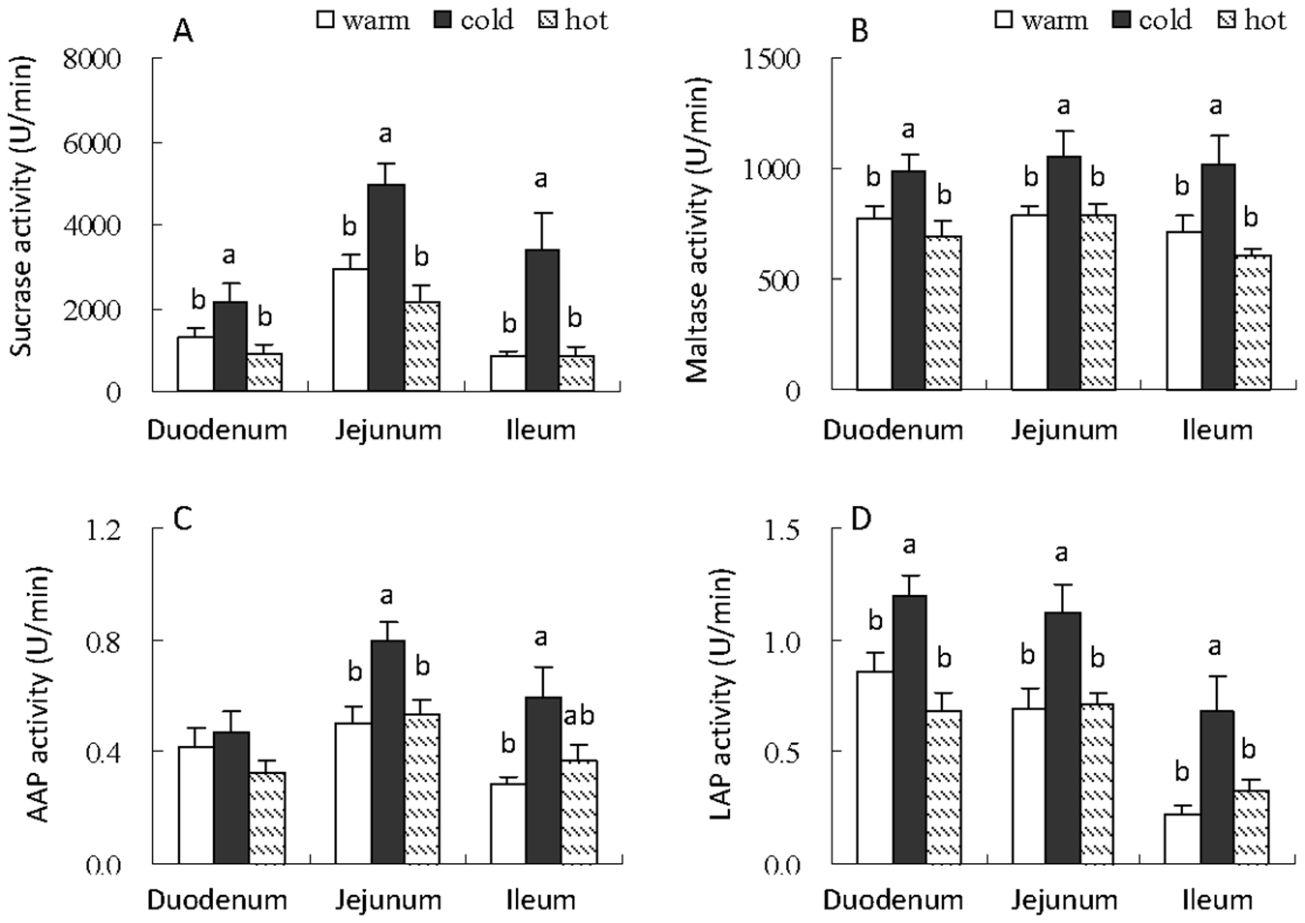
Activity of sucrase (A), maltase (B), AAP (C) and LAP (D) of duodenum, jejunum and ileum in striped hamsters acclimated to warm (23°C), cold (5°C) and hot (31°C) conditions. Different letters (a or b) above the columns indicate significant difference between the three groups (*P*<0.05). Data are means ± s.e.m.

Sucrase activity was different between the three groups, and cold hamsters showed 62.3%, 71.0% and 311.9% higher sucrase activity of duodenum, jejunum and ileum than warm hamsters, and 138.2%, 131.0% and 290.0% higher than hot hamsters (duodenum, *F*_2,22_ = 3.80, *P*<0.05; jejunum, *F*_2,22_ = 12.46, *P*<0.01; ileum, *F*_2,22_ = 8.41, *P*<0.01, Fig. 2A). Consistently, maltase activity was significantly higher in cold hamsters than that in warm and hot hamsters (Fig. 2B). AAP and LAP activity of jejunum and ileum were higher in cold group compared with that in warm or hot group (Fig. 2C, 2D). No significant differences were observed in activities of sucrase, maltase, AAP and LAP of duodenum, jejunum or ileum between warm and hot hamsters (Fig. 2A, 2B, 2C, 2D).

The changes in thermogenesis, energy intake and digestive enzymes, as well as UCP1 gene expression of brown adipose tissue in response to hot-to-cold or cold-to-hot transitions.

### Metabolic rate and maximal nonshivering thermogenesis (NST_max_)

**Figure 3:**
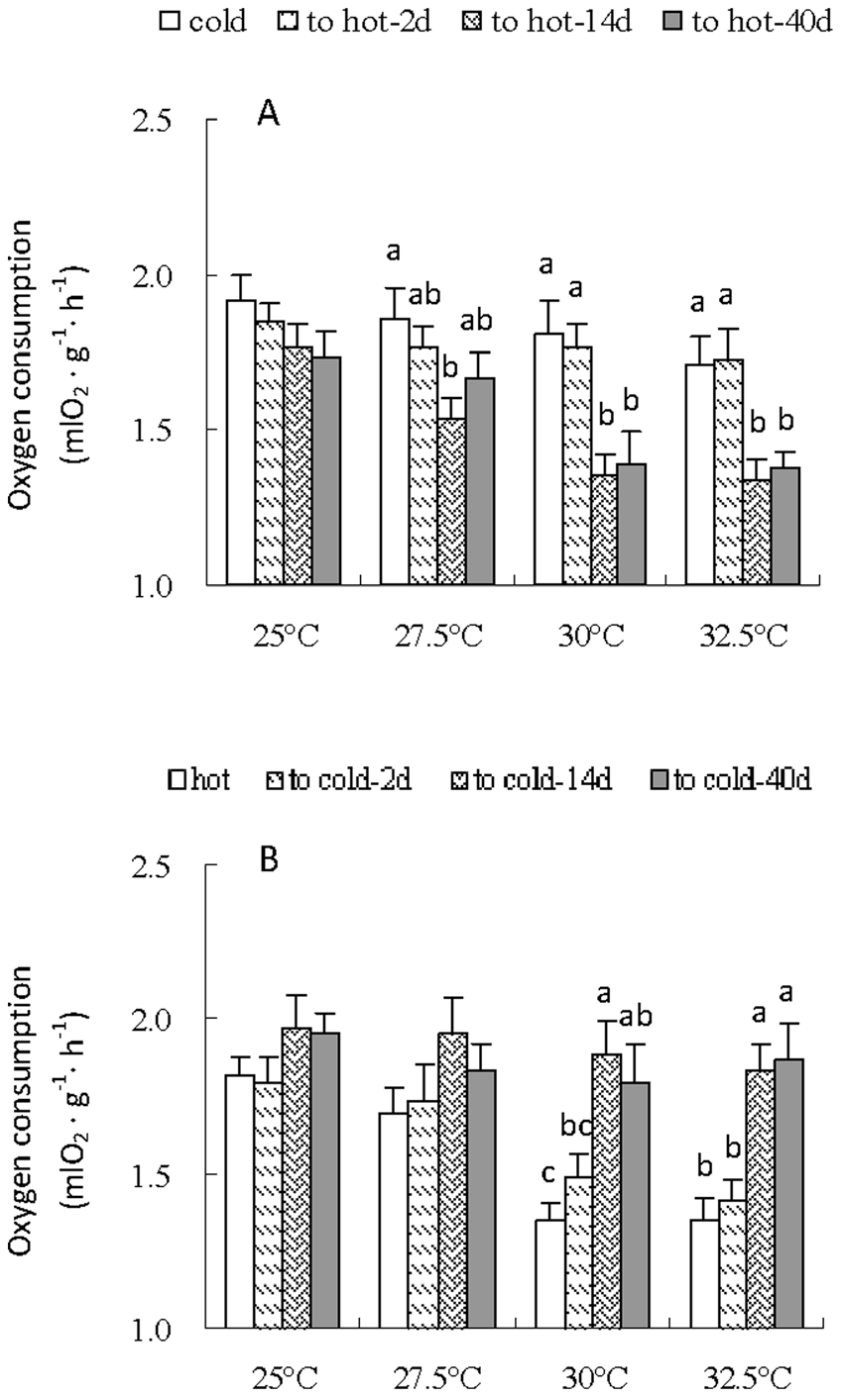
Oxygen consumption of striped hamsters during the transition of cold to hot (A) and hot to cold condition (B). Different letters (a, b or c) above the columns indicate significant difference between the four groups under same temperature (*P*<0.05). Data are means ± s.e.m.

There was no difference in metabolic rate at 25°C between cold hamsters and the hamsters transferred to hot for 2, 14 and 40 days (Fig. 3A). While metabolic rate of to-hot-14d group was significantly lower than that of cold group at 27.5°C (*F*_3,28_ = 2.77, *P* = 0.05). Significant differences in metabolic rate were observed when they were measured at 30 and 32.5°C, and metabolic rate in the to-hot-14d and 40 d groups were lower by 25% and 22% than that in cold groups, respectively (30°C, *F*_3,28_ = 7.28, *P*<0.01; 32.5°C, *F*_3,28_ = 7.22, *P*<0.01). No difference was observed in metabolic rate between warm, to-cold-2d, 14d and 40d groups at 25 and 27.5°C (Fig. 3B). When the hamsters were measured at 30 and 32.5°C, metabolic rate in the hot were significantly lower than that in to-cold 14d and 40 groups, respectively.

**Figure 4:**
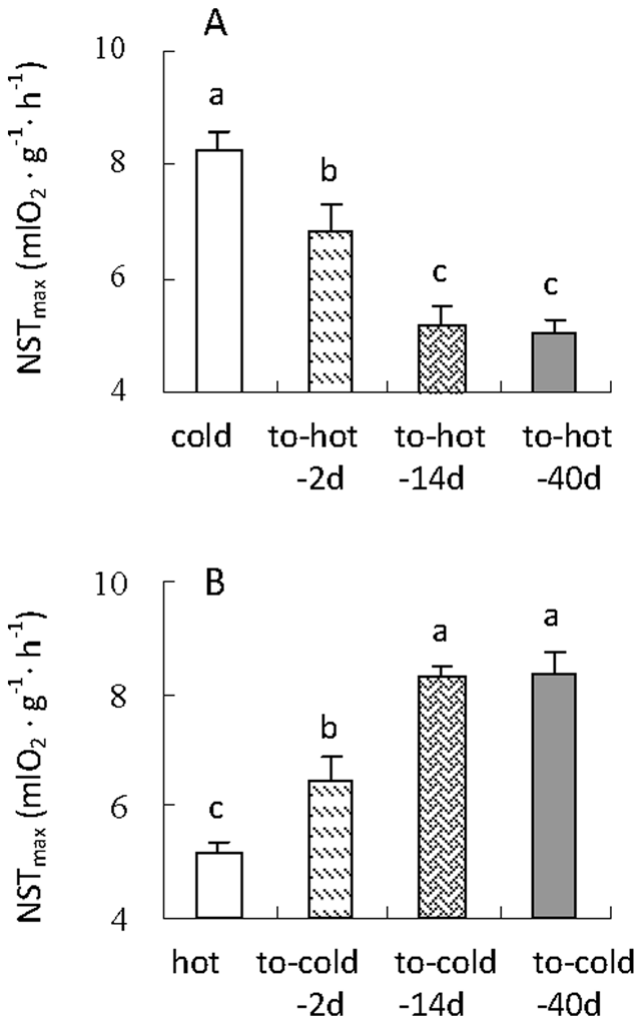
NST_max_ of striped hamsters during the transition of cold to hot (A) and hot to cold condition (B). Different letters (a, b or c) above the columns indicate significant difference between the four groups under same temperature (*P*<0.05). Data are means ± s.e.m.

NST_max_ was different between cold hamsters and those exposed to hot for 2 d, 14 d and 40 d, by which hot transition induced a significant decrease in NST_max_ (*F*_3,28_ = 18.37, *P*<0.01, Fig. 4A). Hamsters in to-hot-2d, 14d and 40d groups showed 17%, 38% and 39% lower NST_max_ than cold hamsters (post hoc, *P*<0.05). The transitions from the hot to cold temperature also had significant effects on NST_max_ (*F*_3,28_ = 23.11, *P*<0.01, Fig. 4B). NST_max_ in to-cold-2d, 14d and 40d groups increased by 26%, 63% and 63% compared with that in hot group, respectively (post hoc, *P*<0.05).

### Energy budget

**Figure 5:**
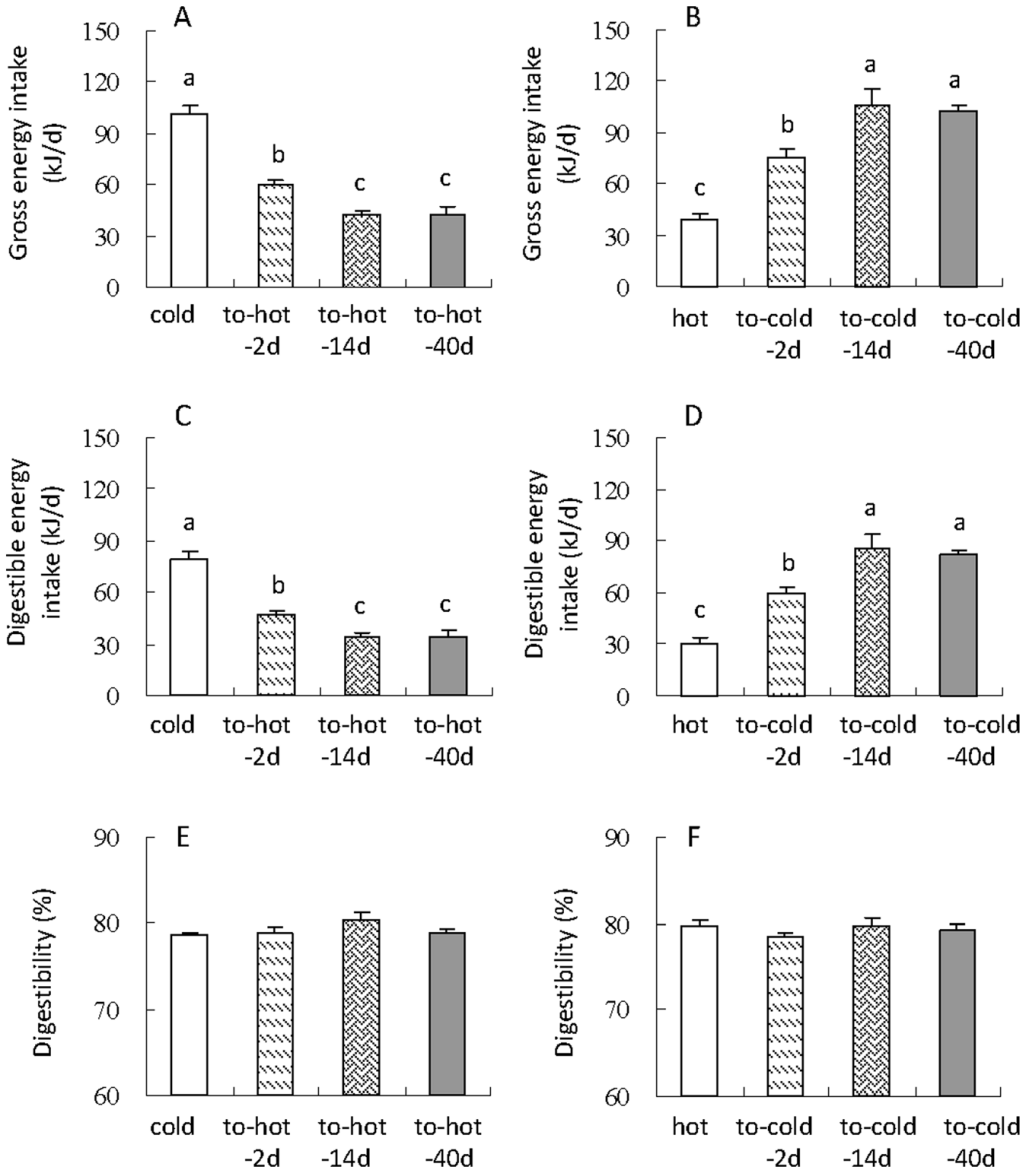
Gross energy intake (A, B), digestible energy intake (C, D) and digestibility (E, F) of striped hamsters during the transition of cold to hot or hot to cold condition. Different letters (a, b or c) above the columns indicate significant difference between the four groups under same temperature (*P*<0.05). Data are means ± s.e.m.

Gross energy intake and digestible energy intake were significantly affected by the transition of cold to hot temperature, by which hamsters decreased both gross energy intake and digestible energy intake after they were transferred from the cold to the hot condition (gross energy intake, *F*_3,28_ = 45.35, *P*<0.01, Fig. 5A; digestible energy intake, *F*_3,28_ = 40.60, *P*<0.01, Fig. 5C). When hamsters were transferred from the hot to the cold temperature, gross energy intake increased by 97%, 175% and 168%, and digestible energy intake elevated by 94%, 176% and 166% compared with that in hot group (gross energy intake, *F*_3,28_ = 28.09, *P*<0.01, Fig. 5B; digestible energy intake, *F*_3,28_ = 25.28, *P*<0.01, Fig. 5D). No difference in digestibility between the cold and to-hot-2d, 4d and 40d groups (Fig. 5E) as well as the hot group and to-cold-2d, 14d and 40d groups (Fig. 5F).

### AAP and LAP activity

**Figure 6:**
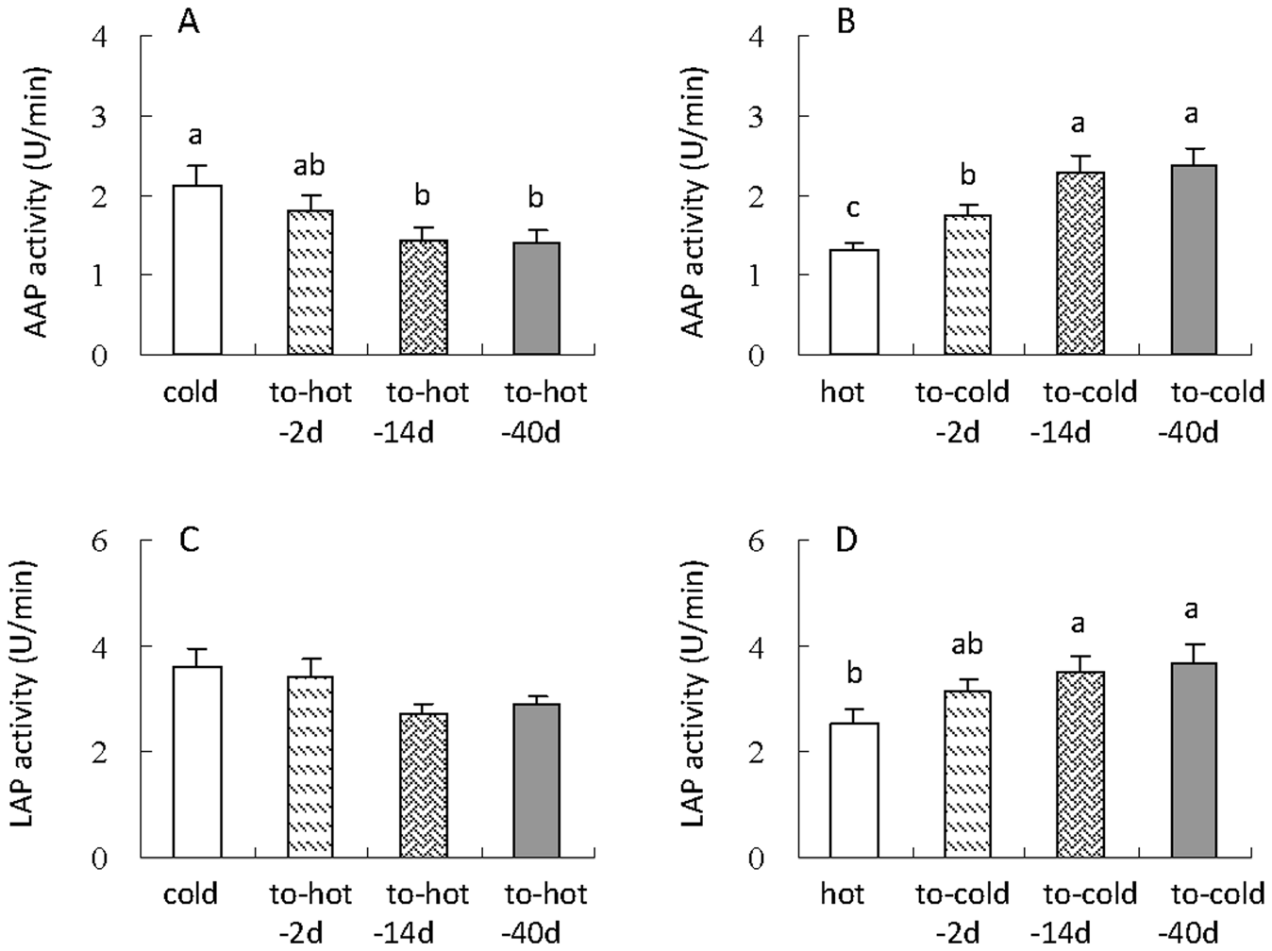
AAP activity (A, B) and LAP activity (C, D) of striped hamsters during the transition of cold to hot or hot to cold condition. Different letters (a, b or c) above the columns indicate significant difference between the four groups under same temperature (*P*<0.05). Data are means ± s.e.m.

AAP activity of small intestine was significantly affected by hot acclimation (*F*_3,28_ = 3.86, *P<*0.05, Fig. 6A), by which the hamsters acclimated to hot temperature for 14 and 40 days showed significant lower AAP activity than hamsters kept at cold. Cold acclimation led to a significant increase in AAP activity (*F*_3,28_ = 9.62, *P<*0.01, Fig. 6B), which was significantly increased in to-cold-14 and 40d groups compared with that in hot group. Inconsistent with AAP, differences in LAP activity between the cold, to-hot-2d, 14d and 40d were not statistically different (*F*_3,28_ = 2.55, *P = *0.08, Fig. 6C). After acclimated to the cold temperature, hamsters showed increases in LAP activity (*F*_3,28_ = 3.14, *P<*0.05, Fig. 6D), by which LAP activity increased by 24%, 37% and 44% in hamsters acclimated to the cold temperature for 2d, 14 and 40d than those maintained at the hot condition.

### UCP1 gene expression of brown adipose tissue

**Figure 7:**
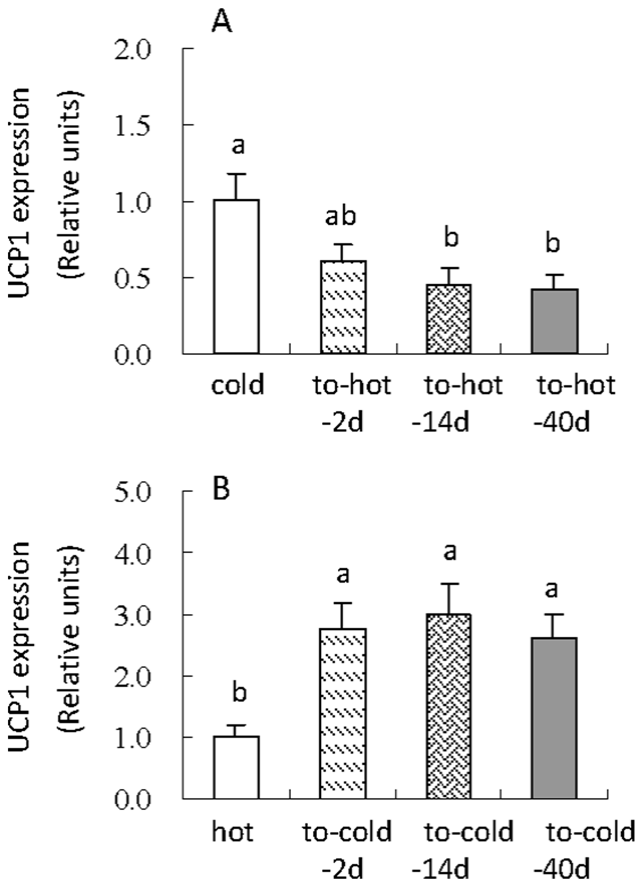
UCP1 gene expression of brown adipose tissue of striped hamsters during the transitions of cold to hot (A) and hot to cold condition (B). Different letters (a, b or c) above the columns indicate significant difference between the four groups (*P*<0.05). Data are means ± s.e.m.

Hot hamsters showed significant decreases in UCP1 gene expression compared with those that were transferred to the cold condition (*F*_3,28_ = 4.66, *P<*0.01, Fig. 7A). UCP1 expression was down-regulated by 39%, 56% and 57% in to-hot-2d, 14d and 40d groups than that in cold group. Cold transition had a significant effect on UCP1 gene expression, by which the hamster that were transferred to cold for 2d, 14 and 40d had 176%, 199% and 161% higher mRNA levels than the animals maintained at the hot condition (*F*_3,28_ = 5.12, *P<*0.01, Fig. 7B).

## Discussion

The thermoneutral zone (TNZ) is one of the most well recognized concepts of thermal physiology of homeothermic organisms, which is previously defined as a range of ambient temperatures where metabolic rate is at basal or resting levels [49]. The method for determining TNZ is based on the oxygen consumption rate, which has been widely used for many decades. The current definition of the TNZ is the range of ambient temperature at which temperature regulation is achieved only by control of sensible heat loss, i.e., without regulatory changes in metabolic heat production or evaporative heat loss (EWL) [50]. EWL comprises passive components (EWL passive), i.e., passive water loss by evaporation as occurs from respiration and water loss through skin, and active EWL (EWL active), e.g., sweating, panting, grooming, moisture on skin [50]. The current definition of the TNZ does not mention the metabolic rate but recommends that defining the upper end of TNZ in terms of the rise in metabolic rate should be avoided. However, it is difficult to pinpoint the threshold temperature where an animal is utilizing ELW passive or ELW active to dissipate heat. In addition, ELW is measured infrequently in metabolic studies, we often must rely on the animal's metabolic rate to assess the TNZ [20].

Romanovsky and his colleague (2002) have developed the methods for determining the TNZ in rats, and proposed three criteria of thermoneutrality [51]. All three criteria of thermoneutrality are determined using core temperature and skin temperature, which are measured using cooper-constantan thermocouples fixed to the base or middle and distal thirds of the tail with tape [51]. They give a narrow range of ambient temperatures and define the TNZ much more precisely, which have been widely used in biomedical research [51]. However, the three criteria of thermoneutrality may not be suitable for the striped hamsters because of very short tail of this species (only 1cm in length). We have to rely on the metabolic rate for the measure of the TNZ in this study.

In the present study, we found TNZ was different between the hamsters acclimated to the cold, warm and hot temperatures, by which the cold- and warm-acclimated hamsters showed a wider TNZ (22.5 or 25 to 32.5°C), similar to that observed in the same strain of hamsters captured in the winter [17]. Here we also found a narrow TNZ in hot-acclimated hamsters (30 to 32.5°C), which was near to that observed in the hamsters that were acclimated to hot summer [17]. Consistently, striped hamsters captured in cold seasons had a wider TNZ and those captured in hot seasons exhibited a narrow TNZ [15], [16].

A classic study on thermoneutrality performed by Scholander and his colleague showed that animals living in the arctic had very low lower critical temperatures and an extremely wide TNZ [18], [20]. Lesotho mole-rat (*Cryptomys hottentotus mahali*) from the cold temperature at high altitude had a lower temperature of the lower critical point and a broader TNZ than animals from the lower altitude [19]. These results suggest that the thermoneutrality of an animal is possibly affected by T_a_. Mammals living in the cold conditions possibly have wider TNZ than those living in hot conditions. Within an animal species, TNZ may shift with the change in T_a_, by which cold acclimation decrease the lower critical temperature and made the TNZ wider, and hot exposure elevated the lower critical temperature, resulting in a narrow TNZ. Thus, this finds would be helpful for considering a temperature range that is suitable for BMR or RMR measurements for the animals acclimated to different T_a_. It has been previously found that seasonal acclimated hamsters show significant variations in weight of pelage and energy budget between summer and winter [14], [17]. Many other mammals also have significantly heavier skin and fur, but show significant increases in metabolic thermogenesis [52], [53], suggesting that insulative properties of integument and metabolic thermogenesis would be involved in the shift of TNZ at different temperatures.

In the present study we observed that metabolic rate increased in striped hamsters when temperature decreased below the lower critical temperature of TNZ. In addition, within TNZ cold-acclimated hamsters showed higher metabolic rate and thermogenesis than those acclimated to hot condition. Similar results have been previously reported in seasonal acclimated striped hamsters [14]–[17] and other wild small mammals, as well as in laboratory rodents [13], [19]–[21], [28], [32], [39], [41], [54]–[57].

It has been suggested that metabolic rate is correlated with the sizes of some visceral organs, including liver, hearts and kidneys which are termed as metabolic machinery [11], [32], [58]–[60]. In the present study, cold-acclimated hamsters had heavier liver, heart and kidneys than those acclimated to the warm and cold temperatures. Cytochrome c oxidase activities of liver, spleen and kidneys significantly increased in cold-acclimated hamsters, but declined in hot-acclimated animals compared with animals maintained at the warm temperature. Again, these results confirmed that animals with relatively higher metabolic rate had relatively large metabolically active machinery [5], [59]–[62].

In comparison with the increased metabolic rate, cold-acclimated hamsters also consumed significantly higher food, and showed higher energy intake than hot- and warm-acclimated animals. In addition, we observed that the masses and the digestive enzymes of small intestine, indicative of the activities of sucrase, maltase, AAP and LAP, increased in cold-acclimated hamsters compared with that in hot- and warm-acclimated animals. The digestive system has been identified as one of the more sensitive systems to the changes in environmental conditions [63]. The adaptive adjustment in the digestive tract including the sizes of the organ and digestive enzymes probably increased the energy processing capacity, which met the increased energy requirement of the animals at cold conditions [5], [64]. Other studies have also demonstrated that cold acclimation results in higher rates of energy intake and hypertrophy of digestive organs [5], [11], [60], [63], [65]–[67]. BMR has been previously reported to be linked to the morphology and enzymes of digestive tracts, as much as 71% of the variation in BMR is explained by the digestive organs in some rodents [60]. Thus, the increases in metabolic rate at cold temperature may be partly due to the increments of morphology and activities of digestive enzymes.

In addition to metabolic rate, we observed significant changes in thermogenesis in striped hamster acclimated to the different temperatures. NST_max_ increased in hamsters acclimated to the cold and decreased in animals exposed to the hot condition, which was in parallel with the changes in cytochrome c oxidase activity and UCP1 gene expression of brown adipose tissue. This was consistent with that observed in the same strain of hamster [11], [14]–[16], [21] and many other small mammals [12], [23]–[25], [27], [32], [41], [44], [48], [54], [55], [68]. This suggests that there may be common mechanisms of thermoregulatory heat production in small mammals through the adaptive regulations in cytochrome c oxidase activity and UCP1 gene expression in response to the environmental temperature change.

More importantly, from the present study we observed that the TNZ shifted in the striped hamsters acclimated to the cold, warm and hot temperatures, which was possibly because of the different lower critical temperature of TNZ between the animals acclimated to the three conditions. It has been proposed that the wide TNZ with low lower critical temperature in cold-exposed animals is partly due to the increment of the rate of metabolism [14], [17]–[20]. As mentioned above we observed higher metabolic rate in striped hamsters acclimated to the cold condition than those acclimated to the hot condition. However, DBA strain of mouse had 33% higher RMR than C3H strain at thermo- neutrality, but their lower critical temperatures were identical [20]. In addition to metabolic rate, cold-exposed animals significantly increased thermogenic capacity [12], [21], [23], [25], [32], [41], [56], suggesting that not only metabolic rate but also the rate of thermoregulatory thermogenesis might be involved in the shift of TNZ. Consistently, Lesotho mole-rat (*Cryptomys hottentotus mahali*) from the high altitude had a lower temperature of the lower critical point, a broader TNZ and greater regulatory non-shivering thermogenesis than animals from the lower altitude [19]. In the present study, we exchanged the hamsters from the cold to hot condition and from the hot to cold condition for two days, and found no significant changes in metabolic rate, indicating that such a transient change in ambient temperature might not lead to the notable shift of TNZ. However, the shift of TNZ did occur when ambient temperature changed longer, indicating that the thermoregulatory characteristics might be associated with the temperatures that animals were acclimated to. The changes in T_a_ likely led to the shift of the lower critical temperature of TNZ. This finding may be helpful for selection of the temperature range for BMR and RMR measurements for the animals undergoing the changes in T_a_. The data from the present study also suggest that shift of TNZ is an important strategy in small mammals in adaption to climate change. Adaptive regulations of digestive enzymes and cytochrome c oxidase activity and UCP1 gene expression of brown adipose tissue are likely involved in the shift of TNZ.

## Summary

In conclusion, TNZ was different between striped hamsters that were acclimated to the different temperatures, by which TNZ was 22.5–32.5°C, 25–32.5°C and 30–32.5°C in the hamsters acclimated to the cold, warm and hot temperatures, respectively. TNZ was likely shifted with T_a_, by which cold acclimation decreased the lower critical temperature and made the TNZ wider, and hot exposure elevated the lower critical temperature, resulting in a narrow TNZ. This was helpful for choosing the temperature range with caution for BMR and RMR measurements for the animals undergoing T_a_ changes. The shift of the lower critical temperature of TNZ is possibly associated the rate of metabolism and thermoenesis, as well as the digestive capacity of gastrointestinal tract at different T_a_. The upper critical temperature of TNZ may be independent of the changes in T_a_. The changes of the lower critical temperature of TNZ are important strategy in adaption to variations of T_a_.
